# Production and evaluation of mono- and di-rhamnolipids produced by *Pseudomonas aeruginosa* VM011

**DOI:** 10.1016/j.dib.2019.103890

**Published:** 2019-04-03

**Authors:** Bhagwan Rekadwad, Vikas Maske, Chandrahasya N. Khobragade, Prajapati S. Kasbe

**Affiliations:** a National Centre for Microbial Resource, National Centre for Cell Science, NCCS Complex, University of Pune Campus, University Road, Ganeshkhind, Pune 411007, India; b Department of Biotechnology, Yeshwant Mahavidyalaya, Swami Ramanand Teerth Marathwada University, Nanded 431602, India; c School of Life Sciences, Swami Ramanand Teerth Marathwada University, Nanded 431606, India; d Department of Pharmacology and Toxicology, National Institute of Pharmaceutical Education and Research (NIPER), Guwahati 781032, Assam, India

**Keywords:** Microbial surfactants, Oil spills emulsification, Remediation technologies, Rhamnolipid, Drug delivery, Drug-loaded nanoparticle emulsion

## Abstract

Rhamnolipids are amphiphilic compounds secreted by bacteria and possess the emulsification ability. Emulsification ability makes microbial surfactants an excellent candidate for assisting in the breakdown and removal of oil spills. Rhamnolipids have been demonstrated for their antibacterial and antifungal activities. This suggests that rhamnolipids play vital roles in the medical, agricultural, bioremediation etc. In the present study, bacterial strain VM011 was isolated from organic farm soil, located nearby Zuari River in Durbhat (Goa, India), where farmlands were irrigated by borewell water. Isolated bacterial strain VM011 was identified as *Pseudomonas aeruginosa* per the Bergey's Manual of Systematic Bacteriology. The Rhamnolipid production ability of *Pseudomonas aeruginosa* VM011 was confirmed using NaCl-methylene blue agar method. Furthermore, rhamnolipid produced by *P. aeruginosa* VM011 emulsify the combustible hydrocarbon such as kerosene (lamp oil). As produced rhamnolipids has an oil-like appearance and consists of two different rhamnolipid confirmed by thin layer chromatography data-di-rhamnolipid with Rf value = 0.16 and mono-rhamnolipid with Rf value = 0.37.

**Table:** Specifications table

Subject area	*Biology*
More specific subject area	*Biotechnology*
Type of data	*Table*
How data was acquired	*Through laboratory experiments using in-house facilities.*
Data format	*Raw, analyzed*
Experimental factors	*As per experimental conditions included in the paper.*
Experimental features	*Wet laboratory work*
Data source location	*National Centre for Cell Science, Pune, India*
Data accessibility	*Included within this article*
Related research article	Following articles may be referred: B. N. Rekadwad, P. K. Ghosh, *Pseudomonas*: a quorum sensing system for improved crop production, in: V. C. Kalia (Eds.), Quorum Sensing and its Biotechnological Applications, Springer Nature, Singapore Pte Ltd., 2018, pp181-191. https://doi.org/10.1007/978-981-13-0848-2_12[1] I. Siegmund, F. Wagner, New method for detecting rhamnolipids excreted by *Pseudomonas* species during growth on mineral agar. Biotechnol. Tech. 5 (1991) 265–268. https://link.springer.com/article/10.1007/BF02438660[2]

**Table:** 

**Value of the data** • The oil-like mixture of two different rhamnolipids possesses excellent emulsifiability which may find application in breakdown and removal of oil spills. • Rhamnolipid produced by Pseudomonas spp. may be used as an alternative source instead of a synthetic chemical component of spraying agents to control fire accidents and subsequent burning of hydrocarbon in Ocean and oil mines. • Rhamnolipid produced by non-pathogenic strain VM011. Therefore, VM011 rhamnolipid may be used for biotechnological exploitations. • Mono and di-rhamnolipid loaded with drug-nanoparticles (NPs) may find application in drug delivery.

## 1 Data

Rhamnolipid were first confined from *Pseudomonas aeruginosa* and portrayed by Jarvis and Johnson in 1949 [3]. These molecules are usually built from the association of rhamnose sugar and hydroxyl (3-hydroxy) unsaturated fats. Rhamnolipid with one sugar particle are alluded to as mono-rhamnolipid, whereas those with two sugar atoms are termed as di-rhamnolipid. In this work, we portray data on production and evaluation of rhamnolipid produced by a non-pathogenic bacterium *P. aeruginosa* VM011 (Table 1). The biotechnological potential and application of rhamnolipid was evaluated by emulsification activity (Table 2).

**Table 1:** Features of rhamnolipid producing strain VM011.

Test	Results	Test	Result
Colony size (mm)	1	Citrate utilization	+
Shape	Circular	Nitrate reductions	+
Margin	Entire	Starch hydrolysis	+
Elevation	Convex	Gelatin hydrolysis	+
Consistency	Non-sticky	Urease	–
Opacity	opaque	Glucose	+
Surface	smooth	Arabinose	+
Cellular morphology	Rod	Xylose	+
Gram nature	–	Lactose	–
Motility	Motile	Sucrose	+
Endospore	Absent	Raffinose	+
Catalase	+	Galactose	–
Oxidase	+	Salacin	–
Indole production	–	Maltose	+
Methyl red	–	Manitol	+
Voges–Proskauer test	–	Glucose	+

**Table 2:** Step-wise observation of rhamnolipid production.

Tests	Reagent used	Observation	Result
NaCl methylene blue agar plate	Methylene blue	Purple blue haze with sharply defined edge around the culture well	Rhamnolipid was produced
Emulsification	n-Hexadecane, Kerosene	Emulsification is observed	Emulsification activity was found.
Extraction and purification	Chloroform and Ethanol (2:1)	Oil like	Rhamnolipid were purified and extracted.
TLC	Chloroform: Methanol: Water (65:15:2)	Colored spot	RF value of sample was found to be (0.16), (0.37)

## 2 Experimental design, materials, and methods

### 2.1 Sampling

**Fig. 1:**
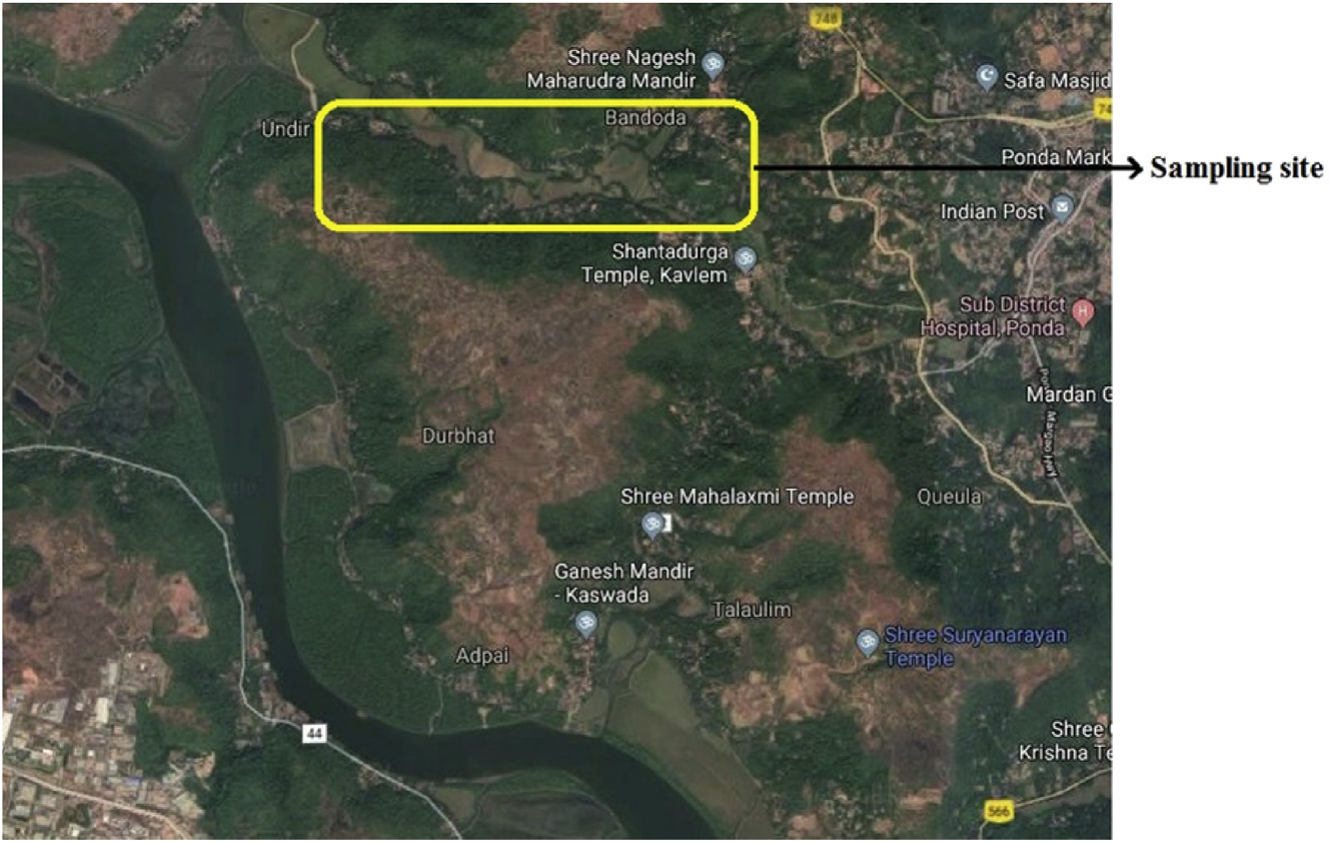
Sampling site: Organic farmland nearby Durbhat in Goa, India.

An organic farm was located near Zuari River in Durbhat (15° 22′N; 73° 58′E), Goa (India). In this area, farmland was irrigated by borewell water. The pre-monsoon soil samples were collected from the sampling site (Fig. 1). The composite sampling method was adopted for the collection of soil sample [4]. Collected soil samples were blackish to red in colour. pH of soil samples was ranged from 7.0 to 7.5.

### 2.2 Media

*Kings agar-* (20g, peptone; 10 ml, glycerol; 1.4g, MgCl_2_; 10g, K_2_SO_4_, 20g, bacteriological agar, 1 L, distilled water; pH 7.2)

*Mineral Salt Medium-* (0.7g, KH_2_PO_4_, 0.9g, Na_2_HPO_4_, 2g, NaNO_3_, 0.4g, MgSO_4_.7H_2_O, 0.1g CaCl_2_.2H_2_O, 2ml, trace element solution; bacteriological agar, 1 L, distilled water; pH 7.2)

*Trace element solution*- (0.2 gm, FeSO_4_.7H_2_O; 1.5g, MnSO_4_. H_2_O, 0.6g, (NH_4_)_6_Mo_7_O_24_).

*Detector solution-* (200 mg/ml, Cetyltrimethyl ammonium bromide/NaCl; 5 mg/ml, methylene blue)

*Key minimal medium*- (0.3%, NH_4_H_2_PO_4_; 0.2%, K_2_HPO_4_, 0.2%, Glucose; 0.5% FeSO_4_; 0.1%, MgSO_4_)

*PPGAS medium-* (0.02 g, NH_4_Cl; 0.02g, KCl; 0.12 g, Tris-HCl; 0.001, MgSO_4_; 1%, protease peptone; 0.5%, Glucose).

*Locating reagent-*glacial acetic acid, sulfuric acid, anisaldehyde (50 : 1: 0.05)

*Stationary phase*- Silica (mesh 70–230)

*Solvent System (Mobile phase)-* Chloroform - methanol-water (65 : 15: 2)

### 2.3 Isolation and identification of the bacterium

#### 2.3.1 Dilution preparation

One gram cleaned soil was suspended in 100 mL sterile MilliQ water. Soil suspension was serially diluted from 10^−1^ to 10^−8^. The dilutions 10^−6^–10^−8^ were used for isolation of bacteria.

#### 2.3.2 Isolation

Rhamnolipid producing bacterial strain VM011 was isolated using the spread plate method. Soil suspension (100 μL) was spread on King's B agar and incubated at 37 °C for 24 h. After incubation, isolated colonies were picked and sub-cultured to get pure culture. Total 14 bacteria were isolated. The pure-cultures of bacteria were preserved at 4 °C in the refrigerator. Biochemical analysis was carried out using a standardized method. Isolated strain VM011 was identified using Bergey's Manual of Systematic Bacteriology and named as *Pseudomonas aeruginosa* strain VM011 [3].

### 2.4 Analytical method

NaCl methylene blue agar plate method was adopted for detection of rhamnolipid production by *Pseudomonas aeruginosa* VM011 [4].

### 2.5 Visualization of rhamnolipid production

Purple blue haze with a sharply defined edge around the culture well was observed [5].

### 2.6 Production and analysis of rhamnolipid

#### 2.6.1 Emulsification activity

Emulsification power of rhamnolipid was measured by vortexing an equal volume of rhamnolipid with kerosene for 1 min. Percentage of the volume occupied by the emulsion was recorded.

#### 2.6.2 Cultivation, extraction and purification

The activated culture of *P. aeruginosa* (24 h, O.D. = 0.5) was inoculated into PPGAS broth (ratio 1:100) and incubated at 37 °C for 24–72 h on orbital shaking at 250 rpm. Bacterial cells were removed by centrifugation (6800 g) from the production medium. The pellet was autoclaved and discarded. The supernatant was acidified using hydrochloric acid (12 M) to attain pH 2.0. Precipitated rhamnolipid was collected by centrifugation at 12,100 g for 30 min. Precipitated rhamnolipid was extracted thrice with chloroform and ethanol (in 2:1 ratio). Cell-free rhamnolipid extract was concentrated in a rotary evaporator so as to get relatively pure rhamnolipid having an oil-like appearance.

#### 2.6.3 Purification of rhamnolipid using thin layer chromatography (TLC)

The silica gel slides were prepared using Silica (mesh 70–230 Merck). Extracted rhamnolipid was spotted at the bottom of the slide by leaving 2 cm distance from the bottom of the slide). Spots were dried using the dryer. Pure rhamnolipid (Sigma-Aldrich) was used as a standard for locating an experimental sample spot. Mobile phase has drawn up the spot via capillary action. After the experiment, the spots were visualized by spraying a developing reagent solution [6], [7].

### 2.7 Calculation of RF value

Formula,

Rf Value = Distance travelled by the solute front (r^1^)/Distance travelled by the solvent front (r^2^)
