# Supplementary material for: Production and evaluation of mono- and di-rhamnolipids produced by Pseudomonas aeruginosa VM011
Source: Data Brief. 2019 Apr 3;24:103890. doi: 10.1016/j.dib.2019.103890 (PMC6461592; doi:10.1016/j.dib.2019.103890)
Supplement: Multimedia component 1 [file mmc1.pdf]

Conflict of Interest and Authorship Conformation Form

Please check the following as appropriate:

- o All authors have participated in (a) conception and design, or analysis and interpretation of the data; (b) drafting the article or revising it critically for important intellectual content; and (c) approval of the final version.

- o This manuscript has not been submitted to, nor is under review at, another journal or other publishing venue.

- o The authors have no affiliation with any organization with a direct or indirect financial interest in the subject matter discussed in the manuscript

- o The following authors have affiliations with organizations with direct or indirect financial interest in the subject matter discussed in the manuscript:

Author's name	Affiliation
Bhagwan Rekadwad	[1]National Centre for Microbial Resource, National Centre for Cell Science, NCCS Complex, University of Pune Campus, University Road, Ganeshkhind, Pune – 411007, India
Vikas Maske	Department of Biotechnology, Yeshwant Mahavidyalaya, Swami Ramanand Teerth Marathwada University, Nanded 431602, India
Chandrahasya N. Khobragade	School of Life Sciences, Swami Ramanand Teerth Marathwada University, Nanded – 431606, India
Prajapati S. Kasbe	Department of Pharmacology and Toxicology, National Institute of Pharmaceutical Education and Research (NIPER), Guwahati – 781032, Assam, India.

Corresponding author
Dr. Bhagwan Rekadwad
